# In-hospital mortality of patients with cardiogenic shock after acute myocardial infarction; impact of early revascularization

**DOI:** 10.1186/s13104-018-3830-7

**Published:** 2018-10-11

**Authors:** Kashif Ali Hashmi, Khawar Abbas, Atif Ali Hashmi, Muhammad Irfan, Muhammad Muzzammil Edhi, Nauman Ali, Amir Khan

**Affiliations:** 1 Chaudhry Pervaiz Elahi Institute of Cardiology, Multan, Punjab Pakistan; 2 Liaquat National Hospital and Medical College, Karachi, Sindh Pakistan; 3 Brown University, Providence, RI USA; 4 Kandahar University, Kandahar, Afghanistan

**Keywords:** Ischemic heart disease, Cardiogenic shock, In hospital mortality, Acute myocardial infarction

## Abstract

**Objectives:**

The purpose of this study was to determine the frequency of in-hospital mortality in 351 patients who developed cardiogenic shock after acute myocardial infarction and by determining this; we might find that how efficiently we could manage this serious condition in our population by knowing the factors which are associated with high mortality after cardiogenic shock. Moreover impact of early revascularization like thrombolytic therapy or angioplasty was also evaluated.

**Results:**

Mean age was 65.41 ± 7.78 years in our study. In-hospital mortality with cardiogenic shock after acute myocardial infarction was found to be 44.73%. Significant association of in-hospital mortality was noted with age, hypertension, diabetes mellitus and BMI. Patients receiving early revascularization were noted to have lower in-hospital mortality compared to those in whom revascularization was not done due to delayed presentation. This study concluded that there is a high frequency (44.73%) of in-hospital mortality in patients with cardiogenic shock after acute myocardial in our population. So, we recommend that for achieving a good outcome and to reduce in-hospital mortality; in addition to rapid diagnosis of this condition, underlying risk factors like hypertension and diabetes should be evaluated and managed accordingly and early revascularization should be done when possible.

## Introduction

Cardiogenic shock is defined as inadequate tissue perfusion due to primary failure of the heart to function effectively [1–3]. Cardiogenic shock is the leading cause of death after acute myocardial infarction (MI). In the absence of aggressive highly experienced technical care, mortality rates among patients with cardiogenic shock are exceedingly high (up to 70–90%). The key for achieving a good outcome is rapid diagnosis, prompt supportive therapy, and expeditious coronary artery revascularization in patients with myocardial ischemia and infarction [4–6]. Evidence of right ventricular dilation on echocardiogram may indicate a worse outcome in patients with cardiogenic shock [7].

A study conducted by Bagai et al., showed that frequency of in-hospital mortality rates in older patients with cardiogenic shock after acute myocardial infarction was 39.1% versus 4.5% in patients without shock [8]. Another study done by Kunadian et al., showed that frequency of overall in-hospital mortality of patients with cardiogenic shock after acute myocardial infarction was 35.5% with no sex difference (male: 35.8% vs female: 35%) [9]. A study conducted at Punjab institute of cardiology Lahore (PIC) showed that frequency of emergency cardiac deaths due to cardiogenic shock was noted to be 74% [10]. Another study showed that frequency of 30 day mortality of patients with cardiogenic shock after acute myocardial infarction was (50.6%) [11].

In the current study, we aimed to evaluate the frequency of in-hospital of patients who develop cardiogenic shock after myocardial infarction and to determine factors which are associated with high mortality. With better understanding of the associated co-morbidities, we might be able to device protocols in loco-regional population to reduce in-hospital mortality in these patients. Moreover impact of early revascularization like thrombolytic therapy or angioplasty was also evaluated.

## Main text

### Inclusion criteria

Patients of cardiogenic shock developing after acute myocardial infarction of age 50–80 years, admitted in Chaudhry Pervaiz Elahi Institute of Cardiology, Multan were included in this study.

### Methods

The duration of the study was from January 2014 till December 2017. After approval from ethical review committee, total number of 351 patients with cardiogenic shock (systolic blood pressure < 90 mmHg for at least 30 min or need for vasopressor to maintain systolic blood pressure > 90 mmHg) after acute myocardial Infarction admitted to the Ch. Pervaiz Elahi institute of Cardiology, Multan, fulfilling the Inclusion/Exclusion criteria were selected. After taking informed written consent, the demographic information like name, age, sex, address and risk factors like diabetes mellitus (DM), hypertension (HTN) and smoking were recorded for each patient. After provision of informed written consent, status of diabetes, hypertension and smoking was assessed. Patients who received early revascularization (thrombolytic therapy or angioplasty) were recorded. BMI was calculated by following formula; BMI = weight in kilograms/height in meters [2]. All the patients were followed during their hospital stay. Frequency of deaths was observed in patients of cardiogenic shock after acute myocardial infarction during their hospital stay.

### Data analysis

Data was analyzed by using statistical package for social sciences (SPSS) version 21. Mean and standard deviation were computed for quantitative variable and frequency and percentage were calculated for qualitative variables. Stratification was done with regards to qualitative variables to see the effect of these modifiers on study groups by using Chi square test. Odds ratio was calculated by medcalc online calculator. Normality was checked through histogram as well as Shapiro–wilk test. *p* value of ≤ 0.05 was considered as significant.

### Results

Out of 351 patients, 246 (70.1%) were male and 105 (29.9%) were female. The mean age of patients was 65.4 ± 7.7. Mean weight and height was 95.3 ± 13.1 kg and 169.8 ± 11.8 cm respectively. Mean BMI was 26.41 ± 7.49 kg/m2. Among 351 patients, 143 (40.7%) patients were found with diabetes mellitus, 231 (65.8%) with hypertension while 137 (39%) patients were obese. Mean duration of diabetes, hypertension and smoking was 16.3 ± 11.7 years, 14.52 ± 9.68 years and 13.87 ± 5.59 years respectively as presented in Table 1. Revascularization was done for 70 (20.5%) patients. In our study, in hospital mortality was found to be 44.7% (157 patients). Stratification was done and post stratification Chi square test was applied. The results showed significant association of in hospital mortality with age (p ≤ 0.0001), diabetes mellitus (p ≤ 0.0001), hypertension (p = 0.029) and BMI (p ≤ 0.0001) while insignificant association was found with gender (p = 0.821). Detailed results of association are presented in Table 2. Significant association of in-hospital mortality was also found with patients receiving revascularization (p = 0.014). Patients without revascularization were found to have high mortality (OR = 0.50) as presented in Table 3.

**Table 1:** Descriptive statistics of study population

	n (%)
Age (years)^a^	65.41 ± 7.78
Hypertension duration (years)	14.52 ± 9.68
Diabetes mellitus duration (years)	16.34 ± 11.65
Smoking duration (years)	13.87 ± 5.59
Weight (kg)	95.26 ± 13.09
Height (cm)	169.81 ± 11.78
BMI (kg/m^2^)	26.41 ± 7.49
Sex	
Male	246 (70.1)
Female	105 (29.9)
Diabetes mellitus	
Yes	143 (40.7)
No	208 (59.3)
Hypertension	
Yes	231 (65.8)
No	120 (34.2)
BMI	
Obese (≥ 30 kg/m^2^)	137 (39)
Non obese (< 30 kg/m^2^)	214 (61)
Revascularization	
Yes	72 (20.51)
No	279 (79.49)

^a^Mean ± SD

**Table 2:** Association of in-hospital mortality with risk factors

	In-hospital mortality n (%)		p value	Odds ratio (95% CI)
	Yes (n = 157)	No (n = 194)		
Sex				
Male	111 (70.7)	135 (69.6)	0.821	1.05 (0.66–1.67)
Female	46 (29.3)	59 (30.4)		
Age group (years)				
≤ 60	35 (22.3)	82 (42.3)	0.000	NA
61–70	73 (46.5)	66 (34)		
> 70	49 (31.2)	46 (23.7)		
Diabetes mellitus				
Yes	91 (58)	62 (32)	0.000	2.93 (1.89–4.54)
No	66 (42)	132 (68)		
Hypertension				
Yes	113 (72)	118 (60.8)	0.029	1.65 (1.05–2.59)
No	44 (28)	76 (39.2)		
BMI				
Obese (≥ 30 kg/m^2^)	84 (53.5)	53 (27.3)	0.000	3.06 (1.96–4.77)
Non obese (< 30 kg/m^2^)	73 (46.5)	141 (72.7)		

Chi square test was applied

p-value ≤ 0.05, considered as significant

**Table 3:** Association of in-hospital mortality with early revascularization

Revascularization	In-hospital mortality n (%)		p-value	Odds ratio (95% CI)
	Yes (n = 157)	No (n = 194)		
Yes	23 (14.6)	49 (25.3)	0.014	0.50 (0.29–0.87)
No	134 (85.4)	145 (74.7)		

Chi square test was applied

p-value ≤ 0.05, considered as significant

### Discussion

In the present study, we found a high frequency of in-hospital mortality (44.7%) in patients that develop cardiogenic shock after myocardial infarction in loco-regional population. We also found that mortality risk was associated with age, diabetes, BMI and hypertension. Moreover, there is a significant impact of early revascularization in reducing mortality in patients developing cardiogenic shock after myocardial infarction.

Cardiogenic shock is the most common cause of death in patients that are hospitalized with acute myocardial infarction [12–14]. Many retrospective analyses have concluded that survival in patients with cardiogenic shock that undergo coronary angioplasty is higher than in patients that do not undergo angioplasty [15–20]. Cardiogenic shock owing to acute myocardial infarction (AMI) portends a poor prognosis [14]. It accounts for the vast majority of deaths in all types of AMI [21, 22]. Literature review revealed that in-hospital mortality after myocardial infarction range from 12 to 85% in different centers [23–29]. The differences are largely due to presence of underlying co-morbidities, risk factors and provision of early aggressive interventions like angioplasty. Moreover, development of cardiogenic shock is the major factor leading to increase in mortality after myocardial infarction.

Zhang et al. [23] found mortality with cardiogenic shock after myocardial infarction in Chinese population to be 83% which is higher compared to all previously described studies. We didn’t find any difference in in-hospital mortality between male and female patients. A study done by Wong et al. [24] concluded that despite differences in patient characteristics including age, history of diabetes and prior CABG between women and men, there was no significant sex difference in in-hospital mortality associated with MI complicated by cardiogenic shock. An extensive review by Vaccarino et al. [30] based on 27 published reports from 1966 through 1994 reported no difference in early mortality between women and men with cardiogenic shock after myocardial infarction.

Age is major factor governing the risk of mortality after myocardial infarction with a higher risk of mortality in patients older than 75 years [24]; on the other hand, early revascularization decreases this risk. A large non-randomized SHOCK Registry demonstrated a markedly lower adjusted risk of in-hospital mortality for those aged 75 years or older that were clinically selected to undergo early revascularization [31]. Singh et al. [11] in their study found increasing age, diabetes mellitus, hypertension and smoking as a strong predictors for in-hospital mortality in patients with cardiogenic shock after myocardial infarction and found a positive relationship between them and mortality rates.

Overall, it was concluded that in-hospital mortality in patients with cardiogenic shock after myocardial infarction is very high; moreover increasing age and co-morbid conditions (hypertension, diabetes mellitus) are very strong predictors of in-hospital mortality after acute myocardial infarction and demands immediate management measures. In the absence of aggressive, highly experienced technical care, mortality rates among patients with cardiogenic shock are exceedingly high (up to 70–90%).

### Conclusion

This study concluded that there is a high frequency (44.73%) of in-hospital mortality in patients with cardiogenic shock after acute myocardial in our population and is associated with increasing age, hypertension, diabetes mellitus and obesity. So, we recommend that for achieving a good outcome and to reduce in-hospital mortality; in addition to rapid diagnosis of this condition, underlying risk factors like hypertension and diabetes should be evaluated and managed accordingly and early revascularization should be done when possible.

## Limitations

The major limitation of the study was that, it represents a single institution data. Moreover, data regarding time delay from onset of symptoms till the patients reached hospital and access to regional health care services was not available. Similarly, history of previous episodes of myocardial infarctions and surgeries including CABG was taken into account and long term follow-up was not done.

## Abbreviations

MI: myocardial infarction
DM: diabetes mellitus
HTN: hypertension
